# Molecular imprinted polymer-based potentiometric approach for the determination of carvedilol and ivabradine hydrochloride in dosage form, spiked human plasma and in presence of their oxidative degradates

**DOI:** 10.1186/s13065-025-01392-7

**Published:** 2025-02-07

**Authors:** Nermine V. Fares, Haitham A. El Fiky, Dina A. Ahmed, Maha F. Abd El Ghany, Amr M. Badawey, Mahmoud A. Tantawy

**Affiliations:** 1 Analytical Chemistry Department, Faculty of Pharmacy, Ain Shams University, Cairo, Egypt; 2 Pharmaceutical Chemistry Department, Faculty of Pharmacy, Future University in Egypt, Cairo, Egypt; 3 Pharmaceutical Analytical Chemistry Department, Faculty of Pharmacy, Cairo University, Kasr El-Aini Street, Cairo, ET-11562 Egypt; 4 Department of Chemistry, Faculty of Pharmacy, October 6 University, 6 of October City, Giza, Egypt

**Keywords:** Carvedilol, Electrode, Imprinted polymer, Ivabradine hydrochloride, Plasma

## Abstract

Carivalan^®^ pharmaceutical formulation, which includes carvedilol and ivabradine hydrochloride, is commonly prescribed for alleviating pain associated with angina. Solid contact ion-selective electrodes with wide range of applications have been developed for analysis of these two active ingredients. Those types of electrodes have common drawbacks. Aside from development of aqueous layer, the incorporated ion exchanger in plasticized membrane is usually unable to differentiate in sensing between two similarly charged lipophilic organic ions. These flaws impeded simultaneous quantification of carvedilol and ivabradine hydrochloride in their dosage form. First, attempts were made to stabilize possible signals by synthesizing hydrophobic multiwall carbon nanotubes-based carbon paste. Precipitation polymerization was used to create molecular imprinted polymers (MIPs) for each drug. MIPs’ graved cavities serve as artificial host-tailored receptors that are able to recognize and bind to individual drugs. Carvedilol MIP-based sensor showed Nernstian slope of 55.30 mV/decade while the corresponding value for ivabradine one was 55.50 mV/decade. The respective LODs were 7.0 × 10^− 8^ M and 6.0 × 10^− 7^ M. Interference from excipients of pharmaceutical formulation, common plasma ions, and possible oxidation byproducts was not witnessed, permitting direct and simultaneous measurement of carvedilol and ivabradine in their tablet solution and spiked human plasma. Furthermore, the proposed technique was compared favorably with the official titrimetric and reported spectrophotometric methods for analyzing carvedilol and ivabradine, respectively.

**Supplementary Information:**

The online version contains supplementary material available at 10.1186/s13065-025-01392-7.

## Introduction

Carvedilol (CAR) (Fig. S1a) has a chemical nomenclature of [1-(9 H-Carbazol-4-yloxy)-3-[[2-(2-methoxyphenoxy) ethyl]amino]propan-2-ol]. It is a nonselective beta (β)-blocker that decreases heart rate and promotes better blood flow. It also dilates blood vessels, functioning like an alpha blocker, which contributes to lowering high pressure [1, 2]. On the other hand, ivabradine hydrochloride (IVA) (Fig. S1b) is chemically described as 3-[3-[[(7 S)-3,4-dimethoxy-7-bicyclo[4.2.0]octa-1,3,5-trienyl]methyl-methylamino]propyl]-7,8-dimethoxy-2,5-dihydro-1 H-3-benzazepin-4-one. It reduces heart rate by specifically and selectively inhibiting the cardiac pacemaker current (If), allowing for direct control of heart rate through sinus node inhibition, all while preserving blood pressure and myocardial contractility [3, 4]. Carivalan^®^ tablets, comprising CAR and IVA, have received a prevalent indication for symptomatic treatment of stable angina. Techniques like High-performance thin-layer chromatography (HPTLC) [5], High-performance liquid chromatography (HPLC) [6, 7] and UV spectrophotometry [8, 9] for investigating CAR and IVA have been detailed with no potentiometric approach for simultaneous determination of the declared drugs.

Potentiometric sensing has emerged as a widely supported analytical podium for measuring different organic and inorganic ions by means of ion-selective electrodes (ISEs) in recent decades [10–13]. Since the performance of ISEs is significantly affected by their ability to selectively respond to the target ion amid potential interferences, considerable advancements have been made in enhancing electrode capability for this discrimination by incorporating either a conventional ionophore or a specially designed molecularly imprinted polymer (MIP) [14, 15]. Ionophores are cage-like structures in which the number of the forming units determines these cages’ sizes. On the other hand, MIPs are intelligent 3D materials having cavities that complement the template drug’s shape and size [16, 17]. Those cavities function as artificial receptors which can distinguish and attach to the target compound via several host-guest interactions, such as; Van der Waals, electrostatic, and hydrogen bonding ones. While molecularly imprinted polymers (MIPs) emulate the molecular discrimination capabilities of antibodies, they offer advantages such as easy customization, user-friendliness, cost-effectiveness, stability under extreme pH conditions, inertness, and compatibility with solvents [18].

They might also be kept and utilized multiple times without losing activity. MIP is synthesized by polymerizing a functional monomer around a template molecule using a cross linking mediator within a porous polymer-creating solvent (porogenic). Following that, the template molecules that form the recognition cavities are removed [19].

Relying on the strong discrimination ability of MIP, an uncomplicated and long-lasting potentiometric analysis was created and verified for the CAR and IVA in their combined tablets, spiked plasma, and in the presence of their oxidative degradates for the first time. This challenging potentiometric approach can detect two lipophilic compounds with comparable positive charges at the same time. Using a solid contact paste composed of graphite and multiwall carbon nanotubes (MWCNTs), two MIP/carbon paste electrodes (CPEs) were created for each drug. Precipitation polymerization was used to independently create the MIPs of CAR and IVA in order to overcome the selectivity problem. Full characterization was then performed to assess imprinting and template drug removal. These sensors were utilized to quantify CAR and IVA concurrently in Carivalan^®^ tablets, human plasma, and in the presence of their oxidative degradates.

## Experimental

The following subsections; (2.1) Apparatus, (2.2) Samples and solvents and (2.3) Preparation of oxidative degradates, are discussed in supplementary material file.

### Synthesis of molecular imprinted polymers for CAR and IVA

The non-covalent precipitation polymerization process was used in this work to manufacture MIPs [20, 21]. Two distinct glass stoppered measuring flasks were filled with 40 mL of DMSO as a porogenic solvent, and 1 mmol of either CAR or IVA was added. For the pre-polymerization complex to self-assemble, 4 mmol of MAA was introduced to each solution, and it was sonicated for ≈ 15 min. Subsequently, 1 mmol of the initiator AIBN and 25 mmol of the cross-linker EGDMA were introduced. The two flasks were cleaned out with N_2_ for approximately ten minutes, and polymerization was then allowed to occur for twenty-four hours at 60 °C in a thermostatic water bath. After two ethanol washes, a 15-minute shaking, and a decantation filter to exclude any unreacted components, the resultant white precipitate was filtered. In order to extract the template, MIP particles were treated with a 9:1, v/v, solvent of methanol: glacial acetic acid. This was done using batch-mode Soxhlet extraction, and the extraction process was tracked using UV/Vis spectrophotometric measurements until the extract solution showed no drug absorbance. Ultimately, MIP was desiccated at 100 °C in an oven after being repeatedly cleaned with distilled water until it reached a neutral pH. Without the template, the matching non-imprinted polymers (NIPs) for both medications were individually created using the previously described procedures. CAR-MIP and CAR-NIP stood for the MIP and NIP for CAR, respectively, while IVA-MIP and IVA-NIP were designated for the synthesized polymers for IVA, in order to maintain consistency throughout the process. Using Differential scanning calorimetry (DSC), Fourier Transform-Infrared (FT-IR), Field Emission-Surface Electron Microscopy (FE-SEM) and Brunauer Emmett Teller (BET) surface area analysis, MIPs’ and NIPs’ morphology was fully characterized. Furthermore, UV spectrophotometry was used to track the produced MIPs’ selectivity and rebinding ability.

### Sensors fabrication

The applied carbon paste was made as previously reported by Afkhami and Ghaedi through combining ≈ 4.3 g paraffin oil with ≈ 10 g carbon powder (consisting of ≈ 2 g MWCNT and ≈ 8 g graphite), which was then carefully placed in a mortar and mixed for ≈ 30 min to produce a uniformly wet paste [22]. To achieve a shiny surface, the paste was pressed firmly into the CPE chamber and polished against filter paper. Eventually, about 10 µL of the membrane cocktail—200 mg PVC, 1 mg TpClPB, 10 mg prepared polymer (MIP or NIP for each analyte) and 0.4 mL NPOE in 5 mL THF—were drop-cast twice to deposit the ion sensing membrane. In order to assess selectivity, CX-4, and CX-6 were used in place of the sensing polymers as examples of common ionophores. Another carbon paste electrode modified with MWCNTS was prepared containing all membrane cocktail without MIP, NIP nor ionophores (CPE). After allowing each electrode to air dry, they were submerged in the appropriate analyte solutions of 1 × 10^− 3^ M in buffer pH 5.0 for a full day.

### Measurements of electrical potential

Separate stock solutions of CAR; 1 × 10^− 3^ M, and IVA; 1 × 10^− 2^ M, were made using BRB, pH 5.0. The same buffer was then used to perform additional dilutions into two different groups of measuring flasks (25 mL), yielding 1 × 10^− 7^ − 1 × 10^− 3^ M and 1 × 10^− 6^ − 1 × 10^− 2^ M concentration ranges for CAR and IVA, respectively. CAR-MIP/CPE and IVA-MIP/CPE sensors were used to perform potentiometric measurements at 25 °C.

Also potentiometric measurement was performed for the other NIP, CPE, CX4 and CX6 based sensors for CAR and IVA. The performance characteristics of the proposed sensors were assessed as per IUPAC guidelines [23, 24]. Regression equations were obtained through creating calibration curves between the measured electric potentials and the logarithmic concentrations.

### Application to Carivalan® tablets

Ten tablets were rumpled and mixed thoroughly. Quantities corresponding to 25 and 10 mg of CAR and IVA, respectively, were carefully transferred to a volumetric flask (25-mL) and diluted to the mark with the corresponding buffer to prepare stock solution of 2.5 × 10^− 3^ M of CAR and 8.5 × 10^− 4^ M of IVA. Potentiometric measurement of this solution was conducted by submerging the two suggested sensors with silver|sliver chloride reference electrode. Each drug’s concentration was calculated with the aid of the matching regression equation.

### Application to spiked plasma

1.0 mL aliquot from CAR stock solution (1 × 10⁻³ M) and 0.5 mL one from IVA stock solution (1 × 10⁻² M) were taken, and transferred into a volumetric flask (10-mL). Two milliliters of human plasma were added, and the mixture was completed to the desired volume using BRB solution, which had been adjusted to a pH of 5.0. After one minute of sonication, electric potentials were measured with the aid of the two suggested electrodes.

## Results and discussion

For the purpose of analysing different hydrophobic drugs in the + ve and/or -ve modes, several ISEs have been constructed [25–28]. Nonetheless, it is always regarded as an analytical challenge to determine the potentiometric response of two lipophilic medications that have the same charge simultaneously. This could be explained by the sensing membrane’s inability to distinguish between various moieties with comparable lipophilicity and electric charges in this case, the doped ion exchanger. The selectivity of ISEs for specific medications over other ions has improved throughout development of ionophores along with their computational based selection; however, distinguishing between similarly charged lipophilic drugs remains a challenge [29].

The purpose of this work was to efficiently use MIP’s high recognition binding capability in order to assess the potentiometric responses of the two co-formulated medicines under study. The doping of carbon paste with carbon nanotubes to address the water layer constraint of solid contact ISE was the first step in the fabrication of two ISEs; CAR-MIP/CPE and IVA-MIP/CPE. The selectivity barrier was also overcome by synthesizing, and integrating MIPs for every medication into the appropriate sensing membrane. Ultimately, the created sensors were used to analyse the pharmaceuticals under study not only in the presence of one another but also in human plasma that has been spiked with both of them and in the presence of their official contaminants.

### Synthesis, optimization and characterization of MIPs

Precipitation polymerization was employed in this work to prepare MIPs that would be employed as molecular recognition elements in the suggested sensing membranes. Low electrical impedance, increased binding sites, and superior dispersion in ion sensing membranes are the characteristic results of this precipitation polymerization approach, producing regularly-seized and -shaped particles [30]. The selection criteria for the functional monomer were based on CAR and IVA chemical structures (Fig. S1a and b), and the presence of basic –NH_2_ groups pointed to the acidic monomer; MAA. Additionally, a detailed investigation of the template drug: MAA ratio revealed that 1:4 was the selected one for polymerization process [31] and electroanalysis. The ability of all polymers to dissolve ingredients in the selected porogenic solvent is one of the essential requirements for MIP synthesis [32, 33]. MIP synthesis of CAR and IVA was carried out using DMSO. The effectiveness of the template recognition, elimination, and rebinding operations in MIP has been thoroughly assessed using a number of methods, as stated below.

#### FT-IR

The FT-IR spectra of CAR and IVA were obtained throughout 4000 –500 cm^− 1^ range, together with their equivalent unleached/leached MIPs [34]. For CAR infrared spectrum, the peaks observed at ≈ 3000 cm^− 1^ was caused by N-H/O-H stretching vibrations. Additionally, the characteristic aromatic carbon = carbon vibrations were observed at ≈ 1650 cm^− 1^, Fig. S2a. The compliance between this figure and the one for its unleashed MIP (Fig. S2b) provides compelling proof of the appropriate imprinting of the CAR template medication in the particles of MIP. Nevertheless, two main alterations were noticed; (1) the sharp peaks were hindered owing to the emergence of hydrogen bonding with MAA’s carboxylic group; and (2) the appearance of aliphatic carbon = carbon bands corresponding to MAA and EGDMA at ≈ 1600 cm^− 1^. In contrast, the leached MIP’s IR spectrum showed no signature IR bands of the CAR template medication, indicating that CAR was effectively removed from the MIP while retaining the distinctive aliphatic polymer bands, Fig. S2c.

Comparably, the presence of IVA’s distinctive infrared bands in its unleached polymer, specifically the aromatic C-H (3000 cm^− 1^), and the aliphatic C = O (1650 cm^− 1^) vibrations, ensured effective imprinting polymerization of the drug IVA as shown in Figs. S3a and b. Furthermore, because MAA is present in the polymer structure, an additional aliphatic carbon = carbon stretching band was detected in the unleached MIP compared to the drug. The absence of IVA drug’s distinctive infrared bands in the leached MIP’s IR spectra provides compelling evidence of the drug’s total elimination as the broad O-H stretching vibration band of MAA appeared at 3200–3500 cm^− 1^ together with the characteristic aliphatic carbon = carbon ones Fig. S3c.

#### FE-SEM

**Fig. 1:**
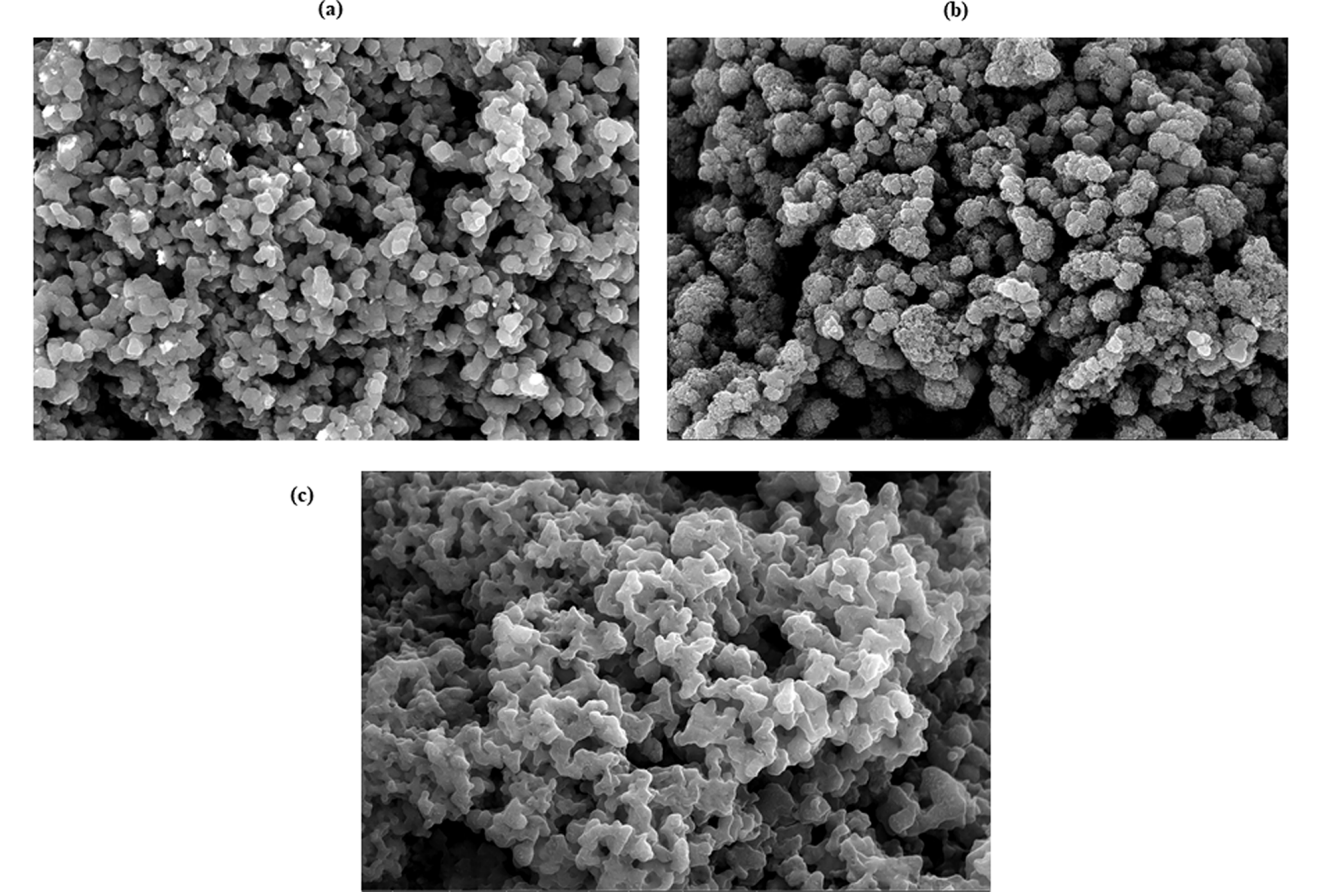
SEM imaging for the prepared MIPs of CAR (**a**), IVA (**b**) and their equivalent NIP (**c**); 10^4^ power of magnification

As seen in Fig. 1, the leached MIP’s and NIP’s surface forms for CAR and IVA have been examined using the FE-SEM method. MIPs along with their matching NIP appeared to have a similar particle size distribution and shape. MIPs display a more rough as well as porous structure compared to NIP’s plain surface, which is indicative of the tiny voids left by the deleted template molecules. The image also showed uniformly sized, spherical beads produced via using the precipitation polymerization process.

#### DSC

**Fig. 2:**
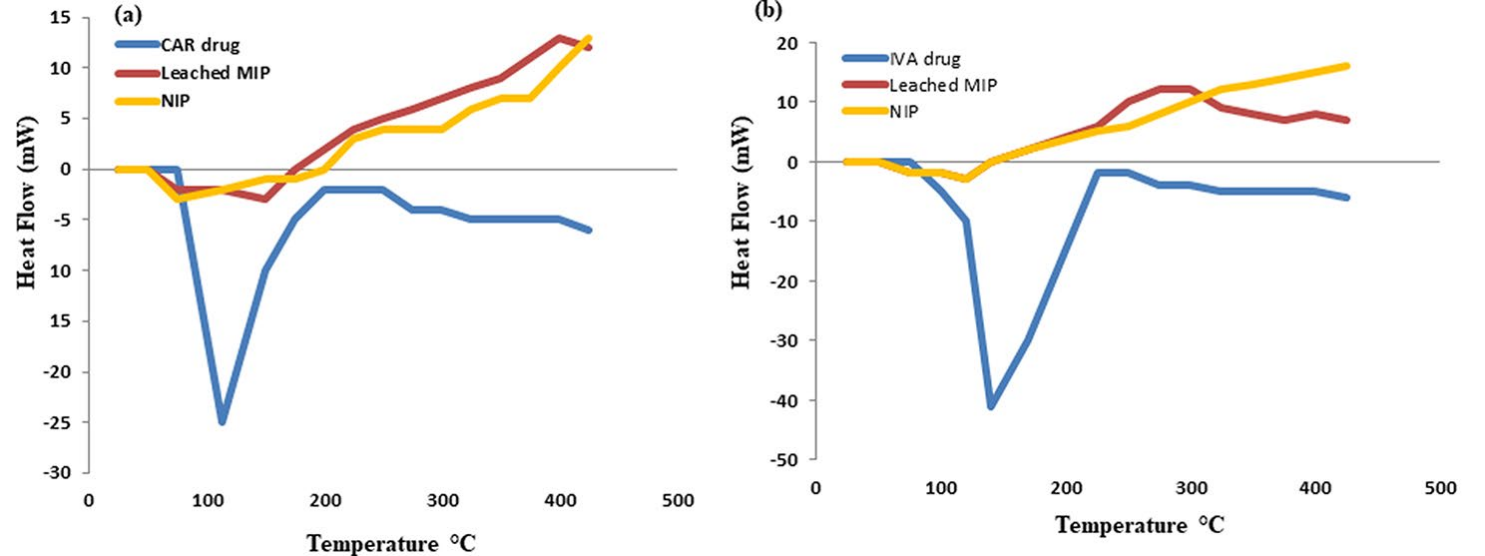
DSC thermograms for CAR (**a**), IVA (**b**) accompanied by their equivalent leached MIPs and NIPs

It is a sensitive and quick thermal examination that determines in what manner a sample’s physical characteristics, e.g., melting point, vary with temperature. In this sense, DSC opens the door to the identification and characterization of a broad variety of compounds, such as medications and polymers. In this work, throughout a 25–425 °C range, thermograms have been obtained for pure CAR and IVA, as well as their corresponding MIP and NIP particles (Fig. 2). For CAR and IVA, a prominent, steep endothermic peak was found at 113 °C and 140 °C, respectively, which correspond to their relative melting temperatures [2, 3]. Additionally, because MIP and NIP had similar structural topologies, their resultant DSC thermograms were equivalent. They demonstrated thermal stability up to 250 °C, at which point exothermic changes took place [35], as seen in Fig. 2. The lack of the previously mentioned drugs’ characteristic peaks in the thermograms of the leached MIPs provided additional evidence of the excellent efficiency of full drugs removal.

#### BET surface area analysis

To determine the porosity extent of the prepared polymers, MIP & NIP, for the two drugs, BET test was carried out. Investigating the polymers’ surface area was essential due to its significant influence on their imprinting functionality. Prior to analysis, any adsorbed moisture was removed from the polymer surfaces by degassing them for four hours at 100° C while nitrogen flow was applied. After that, measurements were made at a temperature of around − 196 °C for liquid nitrogen, and Fig. S4 shows the adsorption/desorption isotherms. The BET equation [36] with a correlation coefficient of at least 0.999 was then utilized to estimate the specific surface area of the polymers using the obtained isotherms. Table 1 runs a summary of all the findings. Pores’ volume and pores’ diameter were additionally computed using the Non Local Density Functional Theory (NLDFT) method [37]. The table showed that the MIPs had a greater surface area and average pores’ volume compared to the corresponding NIPs. The increase in the surface area of MIPs indicated their greater porosity compared to NIPs, as well as the formation of imprinted analyte micro cavities during the polymerization steps. Additionally, results of NLDFT indicated that polymers’ pore diameter exceeded 2 nm, classifying them as mesopores according to the IUPAC definition [38].

**Table 1:** Q values and BET analysis for the prepared polymers

Polymer	Specific surface area (m^2^ g^− 1^)^a^	Pore Volume (cm^3^ g^− 1^)^b^	Average pore diameter (nm)^b^	Binding capacity (Q, µmol g^− 1^)
MIP - CAR	77.78	2.096	6.972	55
NIP - CAR	25.49	1.355	2.126	20
MIP - IVA	66.59	1.161	3.683	57.5
NIP - IVA	22.87	0.858	1.15	22.5

^a^ Specific surface area was calculated using the Brunauer-Emmett-Teller (BET) theory

^b^ Pore volume and diameter were estimated by non-local density functional theory (NLDFT) method

#### Binding capacity and selectivity

Once the template pharmaceuticals have been successfully imprinted in their MIPs and the washing procedure has been completed, a thorough evaluation of each MIP’s re-binding capacity and selectivity is required. Therefore, prior to being integrated as recognition materials in the ISE sensing membrane, the general functionality of the MIP could be ensured. In order to achieve this, spectrophotometric analysis was used to assess MIP and NIP pair’s rebinding affinity to its matching drug (IVA or CAR) along with its discriminating ability for the competing one (CAR or IVA). First, 20 mg of CAR-MIP or CAR-NIP was added to a measuring flask (10-mL) with a 0.05 mM CAR solution in BRB, pH of 5.0, in order to test the MIP-CAR’s rebinding ability. After two hours of stirring, the dispersion was centrifuged for fifteen minutes at 4000 rpm. To find the amount of free drug still present in the supernatant solution, a 0.45 mm Whatman syringe filter was utilized to filter it. The UV absorbance at 285.6 nm was then recorded. For re-binding assessment of the second drug, IVA’s MIP and NIP along with 0.05 mM IVA solutions were utilized as an alternative, the previously indicated methods were tracked to assess the IVA polymers’ rebinding ability, and spectrophotometric plots were recorded at 287.0 nm.

Each polymer’s binding capacity (Q) in µmole g^− 1^ was determined using the next equation:

$$\:Q=\frac{\left({C}_{i}-{C}_{f}\right)\times\:V\times\:1000}{{M}_{Polymer}}$$

Indicating that V is the prepared solution’s volume in mLs, M_polymer_ is the added polymer’s mass in mgs, and C_i_ and C_f_ are the starting and final remained free drug concentrations in mM, correspondingly. Due to the potential formation of indeterminate binding sites throughout the non-covalent polymerization, Q value of each MIP was divided by Q value of its parallel NIP to calculate the imprinting factor (IF). Table 1 illustrates that CAR and IVA’s MIPs had a greater binding capacity than their matching NIP, with computed IFs of 2.75 and 2.56, correspondingly. Large values of IF (> 1) indicate that specific binding is more dominant in the synthesized MIP compared to non-specific binding in the NIP [39]. Subsequently, the produced MIPs’ selectivity was assessed by calculating their binding capacity after being incubated in a solution containing a competing medication at an equivalent dose. For CAR-MIP incubated in 0.05 mM IVA solution, the Q values obtained were 21.9 µmol g⁻¹ for the MIP, while for the IVA-MIP incubated in 0.05 mM CAR, the values were 24.3 µmol g⁻¹. This quick comparison with the previously acquired numbers demonstrated MIP’s selectivity for that particular chemical.

### Incorporation of MIPs

As selectivity modifiers, the produced MIPs were added to the ion-selective membrane. Since the additional MIP indicates the total count of binding sites that are available for identification, which subsequently influences the concentration range the response time and sensitivity of the sensors, it was thoroughly investigated. Many membranes containing 2, 5, 10, 15, 20, and 25 mg of the appropriate MIPs were produced for each of the CAR and IVA drugs, and the electrode response was observed. The response was observed to steadily improve up to 10 mg of MIP, at which point the highest slope and the largest working linearity range were attained. On the other hand, concentrations higher than 20 mg led to an opaque ion-selective membrane and an uneven dispersion of MIP particles. Reduced electrodes’ response and decreased electrical conductance were the results of this. As a result, throughout this investigation, 10 mg MIP was selected for creation of membranes for CAR and IVA MIP/CPEs.

### Performance characteristics of MIP/CPEs

**Fig. 3:**
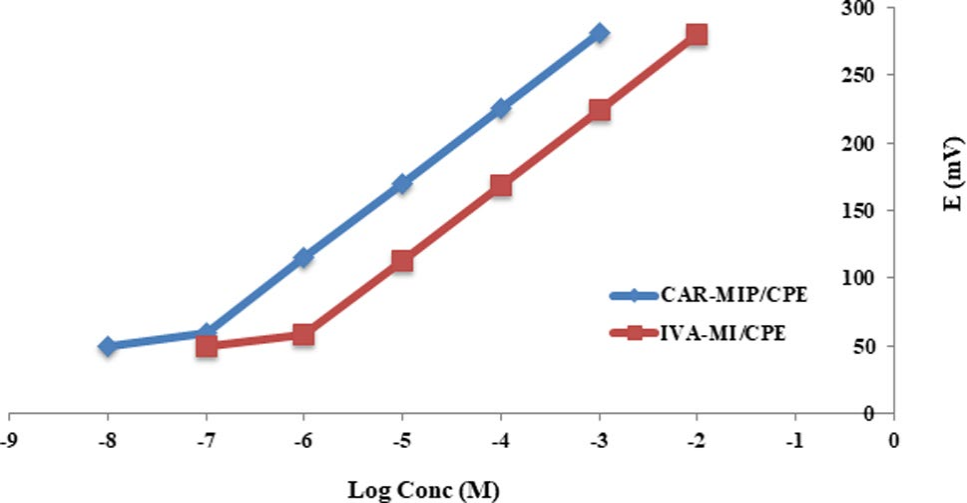
Potentiometric calibrations for CAR (1 × 10^− 7^ M − 1 × 10^− 3^ M) and IVA (1 × 10^− 6^ M − 1 × 10^− 2^ M) using the respective CAR-MIP/CPE and IVA-MIP/CPE

To evaluate the 2 studied sensors’ performance; CAR-MIP/CPE and IVA-MIP/CPE, the existing IUPAC recommendations were adhered to. Plotting two calibration curves, Fig. 3 illustrates an improvement in the linearity concentration of almost compared to the prior values of the respective MIP-free/CPEs with LODs of 7.0 × 10^− 8^ M and 6 × 10^− 7^ M as shown in Table 2. The enhanced performance of the sensors could be ascribed to MIP’s distinct ability to identify the target ions. The sensors’ measurements were determined to have good precision, accuracy, and repeatability based on their low relative standard deviations and satisfactory mean percentage recoveries.

**Table 2:** Potentiometric performance of the proposed electrodes for determination of CAR and IVA

Parameter	CAR					IVA				
	CAR-MIP/CPE	CPE	NIP/CPE	CX-4/CPE	CX-6/CPE	IVA-MIP/CPE	CPE	NIP/CPE	CX-4/CPE	CX-6/CPE
Slope ^a^ (mV/decade)	55.30	45.94	47.396	49.308	49.59	55.50	46.13	45.706	49.782	48.826
Intercept (mV)	446.90	324.10	353.51	386.85	391.86	390.80	283.23	290.77	325.49	331.77
Range (mol L^− 1^)	1.0 × 10^− 7^ – 1.0 × 10^− 3^	1.0 × 10^− 6^ – 1.0 × 10^− 3^	1.0 × 10^− 6^ – 1.0 × 10^− 3^	1.0 × 10^− 6^ – 1.0 × 10^− 3^	1.0 × 10^− 6^ – 1.0 × 10^− 3^	1.0 × 10^− 6^ – 1.0 × 10^− 2^	1.0 × 10^− 5^ – 1.0 × 10^− 3^	1.0 × 10^− 5^ – 1.0 × 10^− 3^	1.0 × 10^− 5^ – 1.0 × 10^− 2^	1.0 × 10^− 5^ – 1.0 × 10^− 2^
LOD (mol L^− 1^) ^b^	7.0 × 10^− 8^	5.0 × 10^− 7^	7.0 × 10^− 7^	9.0 × 10^− 7^	1.0 × 10^− 6^	6.0 × 10^− 7^	9.0 × 10^− 6^	9.0 × 10^− 6^	1.0 × 10^− 6^	1.0 × 10^− 6^
Working pH	2.0–6.0	2.0–6.0	2.0–6.0	2.0–6.0	2.0–6.0	4.0–8.0	4.0–8.0	4.0–8.0	4.0–8.0	4.0–8.0
Response time (s)	5	8	8	7	7	5	8	8	7	7
Stability	45	35	35	40	40	45	35	35	40	40
Correlation coefficient (r)	0.9999	0.9992	0.9992	0.9993	0.9995	0.9999	0.9994	0.9996	0.9996	0.9998
Accuracy ^c^	99.50 ± 1.17	-	-	-	-	100.28 ± 1.34	-	-	-	-
Precision										
Repeatability ^d^	1.268	-	-	-	-	1.041	-	-	-	-
Intermediate precision ^d^	1.445	-	-	-	-	1.516	-	-	-	-
Reproducibility ^e^	1.783	-	-	-	-	1.81	-	-	-	-

^a^ Average of three determinations

^b^ Limit of detection (as per the IUPAC definition, measured by interception of the extrapolated arms of non-responsive and the Nernstian segments of the calibration plot

^c^ Mean ± RSD% of recoveries for five concentration levels measured in triplicate

^d^ RSD% of recoveries for three concentrations of CAR 1 × 10^− 4^, 1 × 10^− 5^ and 1 × 10^− 6^ M) and for IVA (1 × 10^− 3^, 1 × 10^− 4^ and 1 × 10^− 5^ M), each repeated three times within the day for repeatability and repeated in three successive days for intermediate precision

^e^ RSD% of recoveries for two concentrations (1 × 10^− 3^ M and 1 × 10^− 4^ M) measured using three batches of each CPEs

### Influence of various experimental factors

#### pH effect

**Fig. 4:**
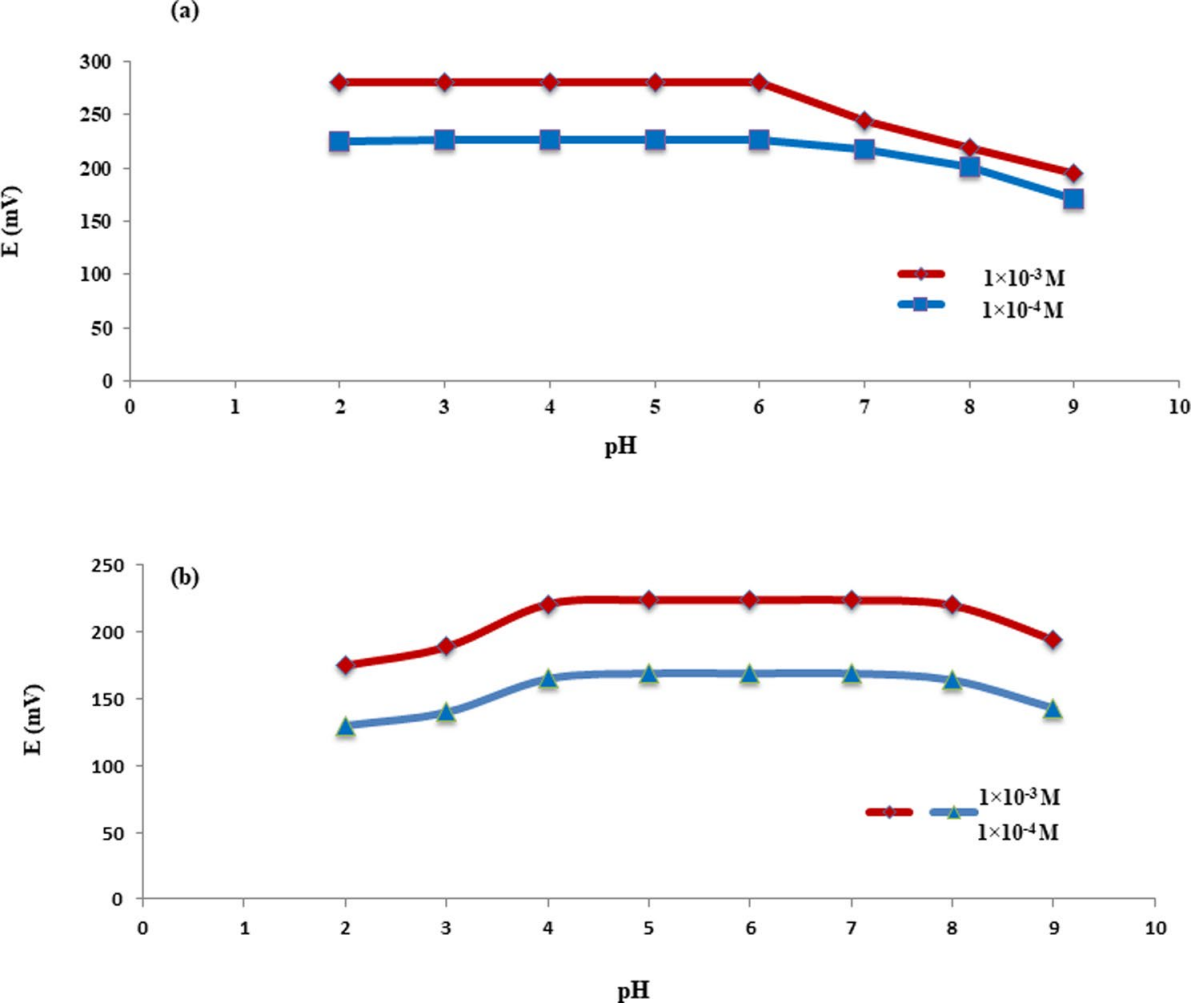
Potential/pH profile for 1 × 10^− 4^ M and 1 × 10^− 3^ M of (**a**): CAR and (**b**): IVA solutions upon using CAR-MIP/CPE and IVA-MIP/CPE, respectively

An investigation into pH has been performed through observing the potential signals of two selected concentrations for each compound across a 2.0 to 9.0 pH range, Fig. 4. For CAR and IVA, a comparatively consistent potential was found within 2.0–6.0 and 4.0–8.0 ranges, respectively. These results coincide with their pKa values; ≈7.7 for CAR and ≈ 9.3 for IVA. Significant potential drift was seen at lower pH values, which may have been caused by the high prevalence of H^+^ ions interfering at those levels. As an alternative, the observed potential gradually decreased at high pH. This could be attributed to the reduced water solubility and the prevalence of molecular drugs on behalf of the ionic ones for CAR and IVA. In order to ensure that CAR and IVA were completely ionized and enable their simultaneous assessment by their respective planned MIP/CPEs, a pH value of 5.0 was used.

#### Soaking time impact

CAR-MIP and IVA-MIP CPEs were immersed in 1 × 10^− 3^ M for varying durations, ranging from 1 h to 36 h, with the aim of assessing the impact of soaking time on the measured response. Construction of calibration curves for each duration period after which the obtained slopes and LODs were compared was the next step. The lengthier the soaking time the better the steady and sensitive electrode response. However, because the electro-active species leached into the soaking solution, continual soaking for longer than 24 h produced undesirable outcomes. The best time to achieve a slope of 50.40 and 50.20 mV/decade for CAR-MIP/CPE and IVA-MIP/CPE, respectively, was determined to be 24 h of soaking.

#### Dynamic response time

The monitoring involved keeping track of the amount of practical time needed for CAR-MIP and IVA-MIP CPEs to achieve stable potential readings (± 1 mV) for every analyte concentration. For the suggested sensors, potential-time curves were created that display around five seconds as the attained response time (Fig. S5). This quick reaction demonstrated how much better ISEs are at quantifying drugs than conventional chromatographic methods.

#### Effect of temperature

With the aid of 1 × 10^− 4^ M standard solutions of each medication, the effects of temperature variations between 25 and 35 °C on the possible way of behaving of CAR-MIP/CPE and IVA-MIP/CPE were evaluated. The recorded responses for both sensors at each temperature are displayed in Fig. S6, indicating that they are practically thermally stable up to 35 °C.

### Sensors selectivity

Potentiometric selectivity coefficients (logK^pot^_D; I_) were estimated using the separate solution method (SSM) [40]. The equivalent NIP sensor (NIP/CPE), Carbon paste electrode (CPE) and two commonly used ionophores (CX-4 & CX-6) based sensors, namely; CX-4/CPE and CX-6/CPE were compared to our proposed MIP/CPE.

**Fig. 5:**
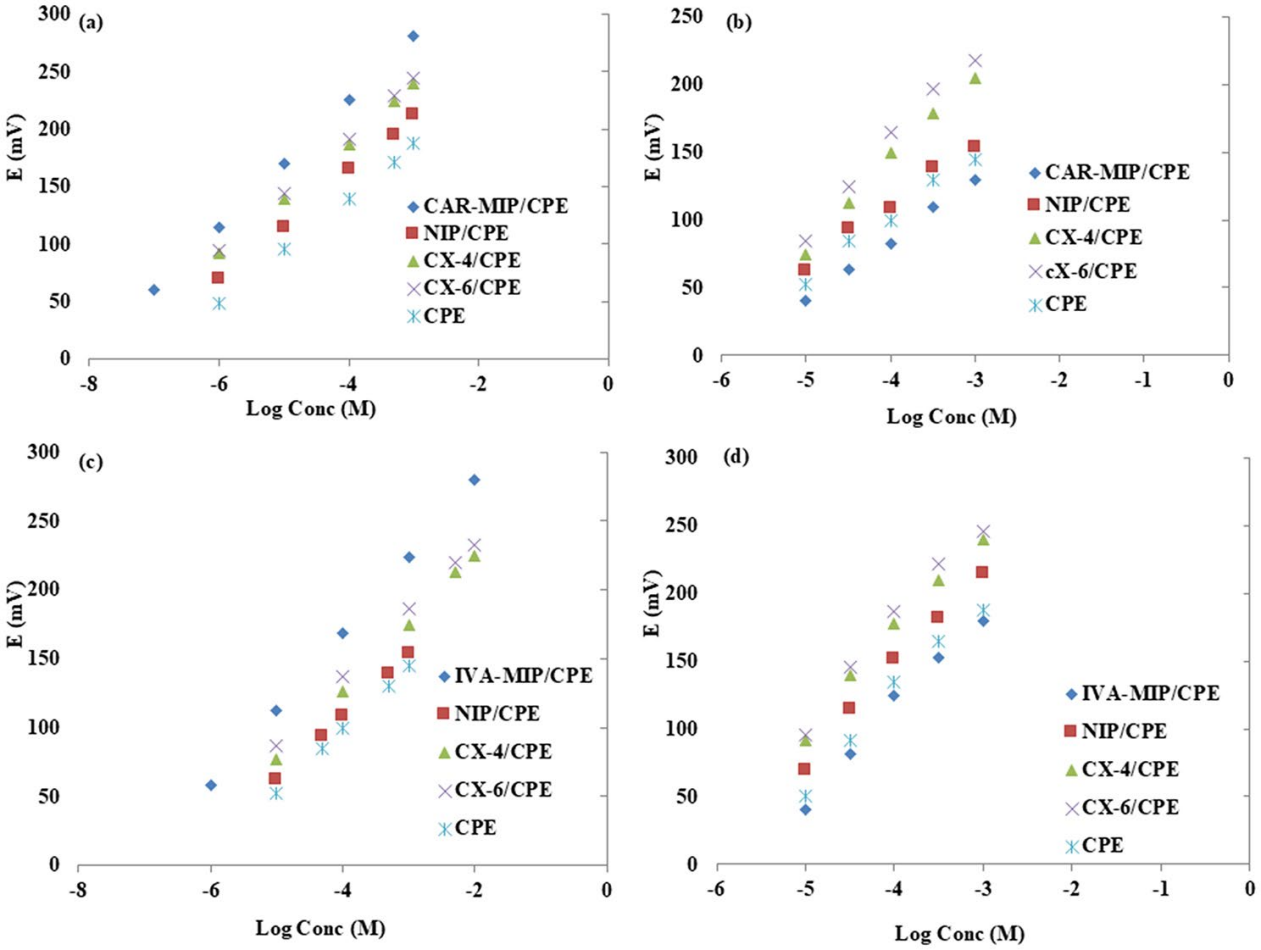
The potentiometric signals of (**a**) different CAR electrodes against logarithms of CAR concentration, (**b**) different as against logarithms of IVA concentration, (**c**) different IVA electrodes against logarithms of IVA concentration and (**d**) different IVA electrodes against logarithms of CAR concentration

To assess the unbiased selectivity of CAR sensors with respect to IVA interference, two calibration curves were constructed for each sensor: one for CAR as the primary ion and another for IVA (Fig. 5a&b). When evaluating the selectivity of IVA sensors against CAR interference, the same procedure was used (Fig. 5c&d).

These figures showed the satisfactory selectivity towards the primary ion with no interference from the other drug. They also showed that the proposed CAR-MIP/CPE and IVA-MIP/CPE have higher range of linearity and better LOD than the other electrodes. Then, for both the primary and interfering drugs, selectivity coefficients were computed at 1 × 10^− 4^ M concentration level. Ionophores-based electrodes, as can be seen, showed minimal improvement. On the other hand, MIP/CPEs outperformed their comparable CPEs or NIP counterparts in terms of enhancing potential selectivity. Following that, a detailed investigation was conducted into the effects of several interfering chemicals regarding the functionality of CAR-MIP and IVA-MIP CPEs. Table 3 provides a summary of the selectivity coefficients, which were established by assessing the potentiometric readings of 1 × 10^− 4^ M for each component. MIP-electrodes demonstrated a high degree of selectivity towards the relevant medicines, with no interference from common inorganic ions like K^+^, Na^+^ or NH_4_^+^. This could be explained by the sensing membranes’ high lipophilicity, which prevented an ionic interchange by inorganic ions. The most popular excipients used in dosage forms and constituents of plasma did not cause any interference either. The outcomes also demonstrated the two sensors’ capacity to avoid possible oxidation byproducts interference and hence could function as stability-indicating.

**Table 3:** Selectivity coefficients based on the separate solutions method (SSM)

Interferent	log (K^pot^_CAR, interferent_) ^a^	log (K^pot^_IVA, interferent_) ^b^
CAR	**0**	**-1.46**
IVA	**-1.70**	**0**
CAR oxidative degradate ^c^	-1.69	-2.15
IVA oxidative degradate ^c^	-2	-1.93
Na^+^	-2.70	-2.76
K^+^	-2.72	-2.79
NH_4_^+^	-3.14	-3.21
Ca^+ 2^	-3.25	-3.35
Mg^+ 2^	-3.48	-3.45

^a^ Average of three determinations using CAR-MIP/CPE

^b^ Average of three determinations using IVA-MIP/CPE

^c^ Oxidative degradation solutions of CAR and IVA

### Application to Carivalan® tablet and spiked plasma

The suggested MIP electrodes were used to quantify CAR and IVA simultaneously in Carivalan^®^ tablet, a recently integrated medicinal formulation. Each sensor’s potentiometric reading was used to calculate the drug’s concentration using its predetermined regression equation. For CAR and IVA, the percent mean recovery was determined to be 100.54 ± 1.20 and 101.54 ± 1.22, respectively. Furthermore, the two sensors were successfully used to analyze CAR and IVA in actual samples of spiked human plasma. The % mean recoveries of CAR and IVA were 99.65 ± 1.986 and 99.57 ± 1.914 respectively. It is worth noting that the respective reported maximum plasma concentrations were around 60 and 20 ng mL^− 1^ [7]. The maximum plasma concentration of CAR is within our studied range while more efforts are still required to enhance the sensitivity of IVA-MIP/CPE. These results underline the potential viability of the suggested electrodes for simultaneous, direct measurement of CAR and IVA without any fear of possible interference from each other, Carivalan^®^ tablet’s excipients, and common plasma ions.

### Statistical comparison & method evaluation

Statistical comparisons of the obtained results with those from the official [1] and reported [8] techniques were conducted using Student’s t-test and F-test for each drug in order to ensure the ability of the suggested electrodes in the assay of CAR and IVA. The obtained values were found to be lower than tabular ones, which is strong proof of an inconsequential difference between the suggested and official procedures, Table S1. A comparative analysis was performed to assess the behavior of the proposed electrodes against other stated potentiometric studies for analyzing CAR [41] or IVA [42–45] as revealed in Table 4. The comparison revealed lower detection limits than some reported ones with wider application in combined tablet, human plasma and in presence of their oxidative degradates achieved by our proposed sensors, thanks to MIP recognition ability. The comparison was also extended to cover other reported techniques for the simultaneous analysis of CAR and IVA [5–9] where the proposed potentiometric method kept its superiority related to wide application and high sensitivity apart from the highly cost-required HPLC/MS technique [7] Table S2.

**Table 4:** Summary of the previously reported potentiometric methods for the assay of CAR or IVA

Ref. No.	Utilized electrode	Analyte	Linearity range	LOD	Application
41	CPE	CAR	3.00 × 10^− 7^ − 1.00 × 10^− 3^ mol L^− 1^	1.5 × 10^− 7^ mol L^− 1^	Single tablet, human plasma and urine samples
42	Liquid contact ISE	IVA	1.00 × 10^− 7^ − 1.00 × 10^− 3^ mol L^− 1^	3.6 × 10^− 8^ mol L^− 1^	Tablet, blood serum and urine samples
43	CPE	IVA	9.80 × 10^− 7^ − 1.00 × 10^− 3^ mol L^− 1^	9.8 × 10^− 8^ mol L^− 1^	Tablet, blood serum and urine samples
44	Liquid contact ISE	IVA	1.0 × 10^− 5^ − 1.00 × 10^− 2^ mol L^− 1^	-	Pharmaceutical formulation and plasma
45	CPE	IVA	1.0 × 10^− 7^ − 1.00 × 10^− 3^ mol L^− 1^	3.6 × 10^− 8^ mol L^− 1^	Pharmaceutical formulation
This work	MIP/CPEs	CAR	1.00 × 10^− 7^ − 1.00 × 10^− 3^ mol L^− 1^	7 × 10^− 8^ mol L^− 1^	Combined dosage form, human plasma and in presence of degradation products
		IVA	1.00 × 10^− 6^ − 1.00 × 10^− 2^ mol L^− 1^	6 × 10^− 7^ mol L^− 1^	

## Conclusion

This study presented a first potentiometric method for assaying CAR and IVA drugs simultaneously. The method demonstrated its efficacy in resolving the probable nosiness which traditionally impeded the potentiometric assessment of two drugs with identical charges and lipophilic characteristics. The strong identification capability of MIPs, made especially for each medicine, served as the foundation for the main idea. A number of characterization methods, such as UV spectrophotometric analysis, DSC, BET, FT-IR, and FE-SEM, guaranteed the success of the templates’ imprinting, removal, and rebinding processes in the manufactured MIPs. Two ion-selective electrodes were created by independently incorporating the produced MIPs into the sensing membranes. The results demonstrated the potential contribution of MIPs in improving selectivity. The added MIP improved the efficiency of ISEs not just when examining the two drugs in question together but also while examining them in the presence of their oxidative degradates. Without any interference from organic or inorganic excipients, the suggested potentiometric sensors were applied to combined tablet and spiked plasma samples with success. In a very brief statement, the goal of this work was to shed more insight into the potential uses of MIPs in the potentiometric examination of medications with similar charges.

## Electronic supplementary material

Below is the link to the electronic supplementary material.

Supplementary Material 1

## Data Availability

The datasets used and/or analysed during the current study are available from the corresponding author on reasonable request.

## Abbreviations

AIBN: Azobisisobutyronitrile
BET: Brunauer Emmett Teller
CAR: Carvedilol
CPE: Carbon paste electrode
CX-4: Calix[4]arene
CX-6: Calix[6]arene
DSC: Differential scanning calorimetry
EGDMA: Ethylene glycol dimethacrylate
FE-SEM: Field Emission-Surface Electron Microscopy
FT-IR: Fourier Transform-Infrared
HPLC: High-performance liquid chromatography
HPTLC: High-performance thin-layer chromatography
ISE: Ion-selective electrode
IVA: Ivabradine hydrochloride
MAA: Methacrylic acid
MIP: Molecularly imprinted polymer
NIP: Non-imprinted polymer

## Declarations

### Generative AI and AI-assisted technologies in the writing process

During the preparation of this work the author used [Chat GPT] in order to rephrase and grammar checking of the paper. After using this tool, the author reviewed and edited the content as needed and take full responsibility for the content of the published article.
