# Supplementary material for: Molecular imprinted polymer-based potentiometric approach for the determination of carvedilol and ivabradine hydrochloride in dosage form, spiked human plasma and in presence of their oxidative degradates
Source: BMC Chem. 2025 Feb 7;19(1):32. doi: 10.1186/s13065-025-01392-7 (PMC11806558; doi:10.1186/s13065-025-01392-7)
Supplement: Supplementary file 1 — Supplementary Material 1 [file 13065_2025_1392_MOESM1_ESM.docx]

**Supplementary Material**

**for**

**Molecular imprinted polymer-based potentiometric approach for the determination of carvedilol and ivabradine hydrochloride in dosage form, spiked human plasma and in presence of their oxidative degradates**

Nermine V. Fares^a^, Haitham A. El Fiky^b^, Dina A. Ahmed^b^, Maha F. Abd El Ghany^a^, Amr M. Badawey^c^, Mahmoud A. Tantawy^c, d^

^a^ Analytical Chemistry Department, Faculty of Pharmacy, Ain shams University, Cairo, Egypt.

^b^ Pharmaceutical Chemistry Department, Faculty of Pharmacy, Future University in Egypt, Cairo, Egypt.

^c^ Pharmaceutical Analytical Chemistry Department, Faculty of Pharmacy, Cairo University, Kasr El-Aini Street, ET-11562, Cairo, Egypt.

^d^ Department of Chemistry, Faculty of Pharmacy, October 6 University, 6 of October City, Giza, Egypt.

**2.1. Apparatus**

A Thermo Scientiﬁc Silver/silver chloride reference electrode (USA), a Jenway pH electrode (UK). A Jenway pH meter (UK) for pH and potential measurements. The working electrode was a carbon paste electrode model MF-2010 (BASi, USA). Shimadzu double beam spectrophotometer (UV, Japan). A water bath (South Korea). Soxhlet apparatus (USA). IR Spectrophotometer Shimadzu (Japan). Differential scanning calorimeter DSC (Japan). Field emission scanning electronic microscope model FEG (USA). Brunauere-Emmette-Teller (BET) analyzer from Microtrac (Japan) were employed in characterization.

**2.2. Samples and solvents**

Pure samples of CAR and IVA were generously provided by Global Napi, located in 6th of October City, Egypt. The purity of the samples was verified as 99.88 ± 0.95 and 99.60 ± 1.06, respectively, based on both official **[1]** and previously reported **[8]** methods. The commercially available Carivalan® tablets, manufactured in France by Les Laboratories Servier Industrie, were also acquired. According to the product label, batch number 29044 of Carivalan® contains 12.5 mg of CAR and 5.0 mg of IVA. All chemicals and reagents used were of analytical grade. These included methacrylic acid (MAA), ammonium persulfate (APS), methanol, graphite powder (20 mm), tetrahydrofuran (THF), calix [4]arene-25,26,27,28-tetrol (CX-4), aniline, sodium dodecyl sulfate (SDS), calix [6]arene (CX-6), potassium tetrakis (4-chlorophenyl) borate (TpClPB), chloroform, ethylene glycol dimethacrylate (EGDMA), 2-nitrophenyl octyl ether (NPOE), 30% hydrogen peroxide (H_2_O_2_), azobisisobutyronitrile (AIBN), glacial acetic acid, dimethylsulfoxide (DMSO), high molecular weight polyvinyl chloride (PVC), ethanol, paraffin oil, multiwalled carbon nanotubes (MWCNTs); ≥ 98% C content, I.D.×O.D.×L≈4.5×10 nm×36 mm). All items were sourced from Sigma Aldrich (Germany). 40 mM from each of boric, phosphoric and acetic acids were mixed together for preparing Britton-Robinson buffer (BRB), and then 0.2 M NaOH was used for adjusting the pH (ranging from 2.0 to 9.0). Human plasma was purchased from (VACSERA, Egypt).

**2.3. Preparation of oxidative degradates**

In two round flasks, measured quantities of CAR and IVA were each refluxed with 10 mL of 30% hydrogen peroxide (H_2_O_2_) for 8 hours at 80°C. After the reflux process, the remaining hydrogen peroxide was evaporated using a hot plate set at 50°C. All oxidative degradates were prepared at a concentration corresponding to 1.0 × 10⁻⁴ M of its parent drug, using Britton-Robinson buffer (BRB) as the solvent.


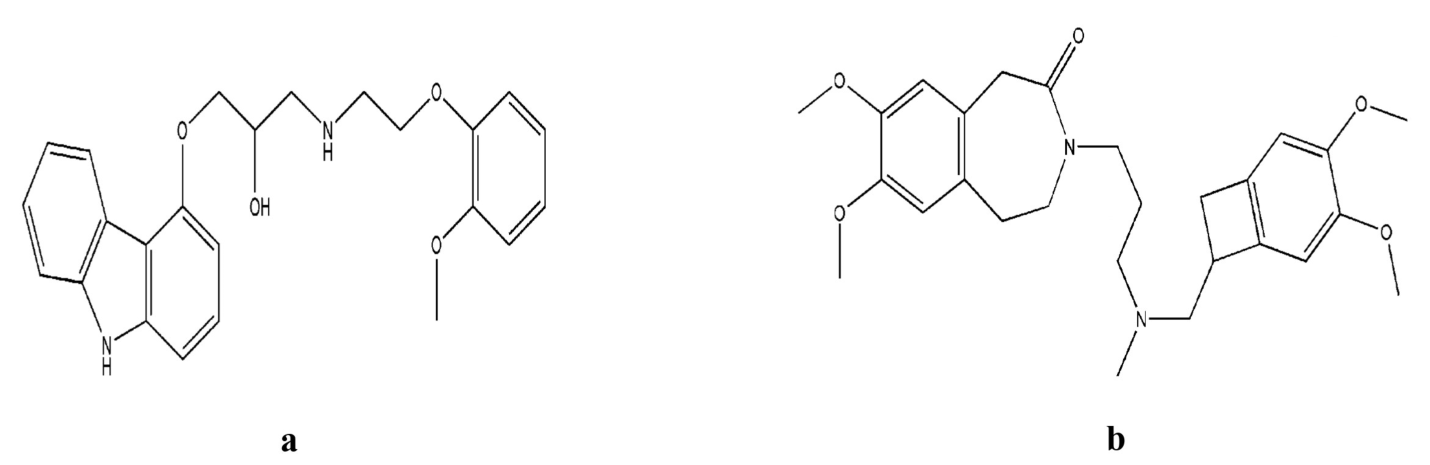


**Figure S1.** Chemical structures of (a) Carvedilol and (b) Ivabradine.


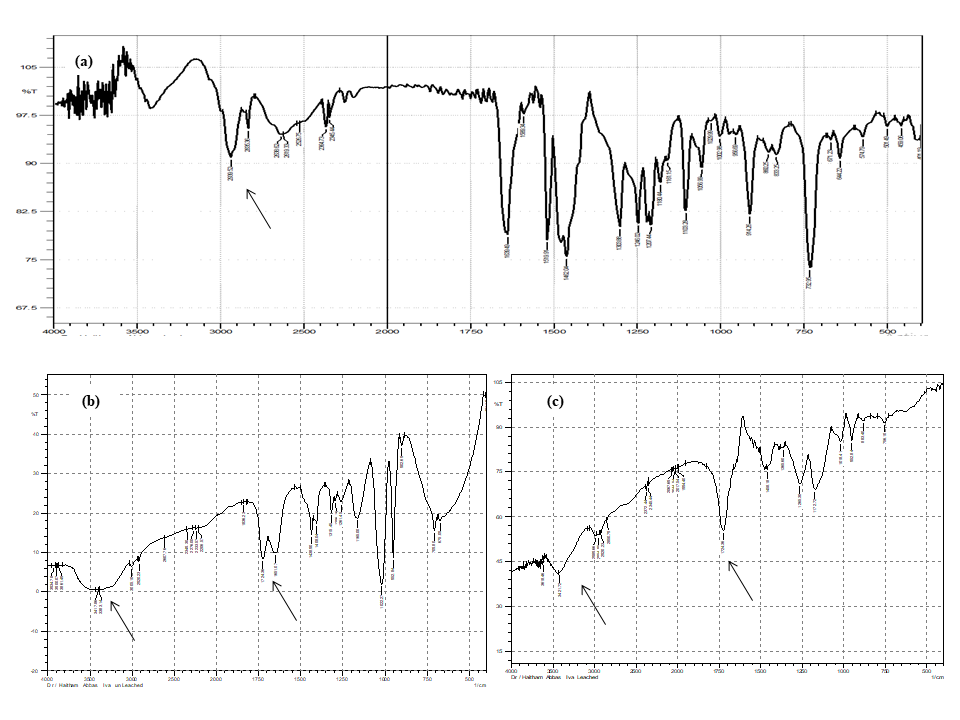


**Figure S2.** IR spectra of CAR (a), its corresponding unleached MIP (b) and leached MIP (c).


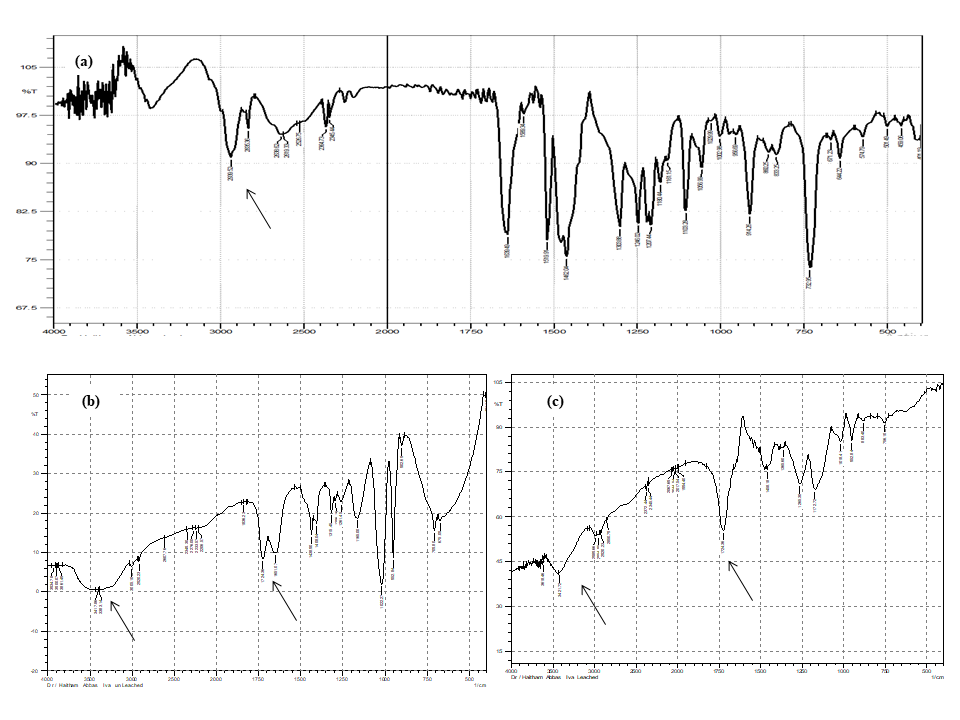


**Figure S3.** IR spectra of IVA (a), its corresponding unleached MIP (b) and leached MIP (c).


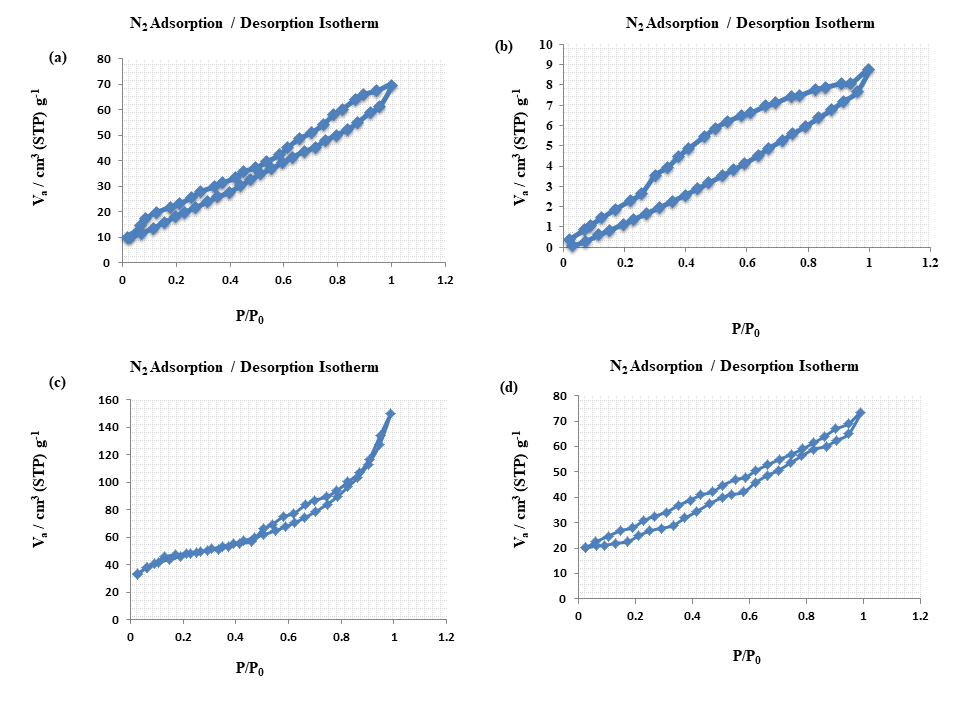


**Figure S4.** The obtained nitrogen adsorption/desorption isotherms for (a): MIP of CAR, (c): MIP of IVA and their corresponding NIPs (b & d, respectively).


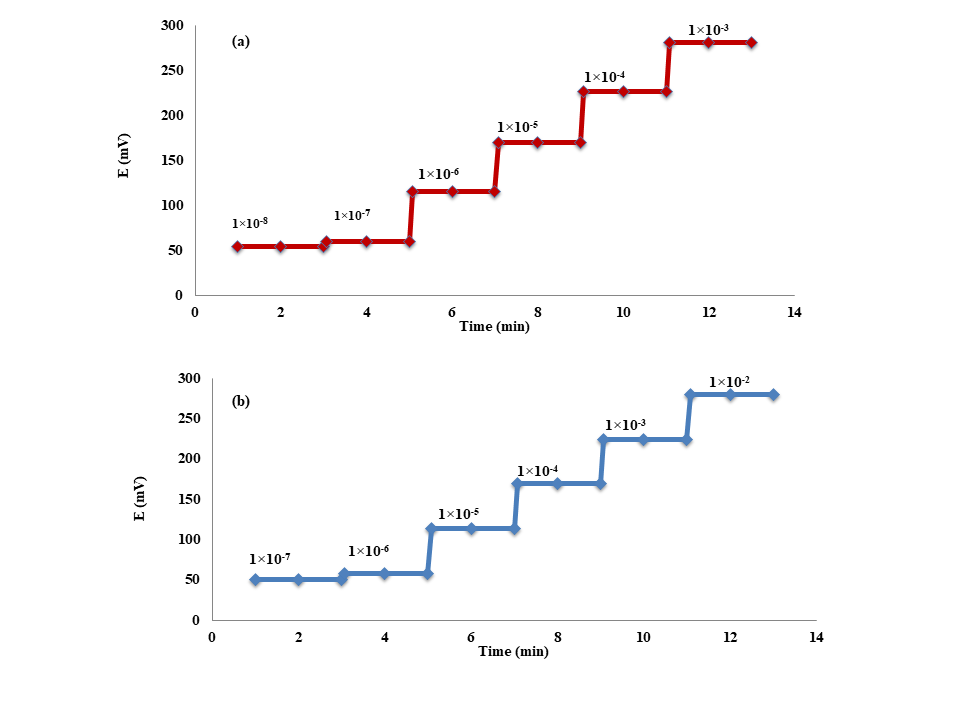


**Figure S5.** Plot of potential as a function of time for responses of (a): CAR-MIP/CPE and (b): IVA-MIP/CPE towards increasing concentrations of corresponding drug solutions.

**
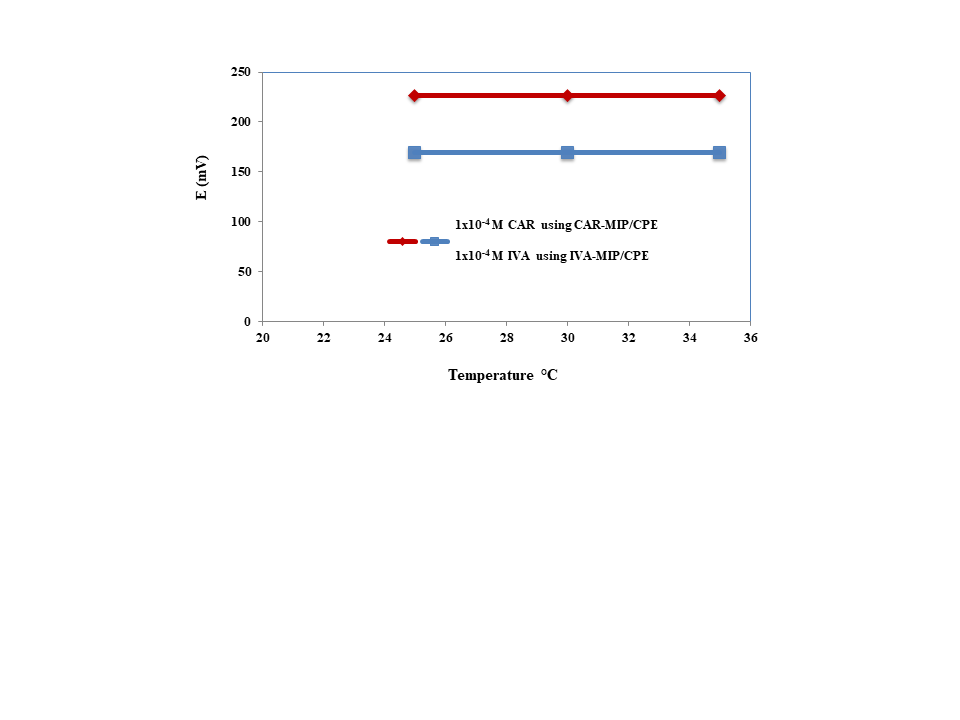
**

**Figure S6.** Effect of temperature on the response of CAR-MIP/CPE and IVA-MIP/CPE.

**Table S1.** Statistical comparison between the proposed potentiometric method, the official and the reported ones for the analysis of CAR and IVA.

| **Parameter** | **CAR-MIP/CPE** | **Official method ^a^** | **IVA-MIP/CPE** | **Reported method ^b^** |
| --- | --- | --- | --- | --- |
|  |  |  |  |  |
| **Mean** | 99.50 | 99.88 | 100.28 | 99.60 |
| **SD** | 1.17 | 0.95 | 1.34 | 1.06 |
| **n** | 5 | 4 | 5 | 5 |
| **Variance** | 1.37 | 0.90 | 1.80 | 1.12 |
| **Student^'^s *t* test ^c^** | 0.761 (2.365) |  | 0.634 (2.306) |  |
| **F ^c^** | 1.522 (15.10) |  | 1.607 (9.60) |  |

^a^ Official potentiometric titration method for determination of CAR against perchloric acid..

^b^ Derivative ratio spectrophotometric method for determination of IVA; peak amplitudes measured at 255 nm.

^c^ The values in parentheses represent the corresponding tabulated values of t and F at p=0.05.

**Table S2.** An overview on the reported methods for the simultaneous analysis of CAR and IVA.

| **Ref. No.** | **Applied technique** | **Detection limit for CAR** | **Detection limit for IVA** | **Application** |
| --- | --- | --- | --- | --- |
| **5** | HPTLC | 102.24 ng band^-1^ | 115.34 ng band^-1^ | Laboratory prepared mixtures |
| **6** | UPLC | 25.0 µg mL^-1^ | 8.9 µg mL^-1^ | Tablet and in presence of degradation products |
| **7** | HPLC/MS | 0.1 ng mL^-1^ | 0.1 ng mL^-1^ | Rats’ plasma |
| **8** | UV spectrophotometry | 0.815 µg mL^-1 a^ | 0.628 µg mL^-1 a^ | Tablet |
| **9** | UV spectrophotometry | 0.258 µg mL^-1 a^ | 0.204 µg mL^-1 a^ | Tablet and in presence of degradation products |
| **This work** | Potentiometry | 7.0 × 10^-8^ mol L^-1^ | 6.0 × 10^-7^ mol L^-1^ | Combined dosage form, human plasma and in presence of degradation products |

^a^ The lowest value was selected among the different approaches applied.
